# Supplementary material for: The genome sequence of the Corsican Wall Brown, Lasiommata paramegaera (Hübner, 1824) (Lepidoptera: Nymphalidae)
Source: Wellcome Open Res. 2026 Sep 14;11:666. [Version 1] doi: 10.12688/wellcomeopenres.27821.1 (PMC13743842; doi:10.12688/wellcomeopenres.27821.1)
Supplement: Peer review history

# Reviewer report: Reviewer response for version 1

**DOI:** 10.21956/wellcomeopenres.30614.r169084

**Published:** 2026-11-06

**Authors:** Virpi Ahola

**Affiliations:** 1 University of Eastern Finland, Kuopio, Kuopio, Finland

In this study, the authors report the genome sequence of *Lasiommata paramegaera*. The work is part of the Project Psyche initiative, which aims to sequence the genomes of European Lepidoptera species. The background and motivation for the study are well described. Long-read PacBio sequencing combined with Hi-C data was used to assemble two haplotypes. The assembly was carefully curated and resulted in a high-quality genome assembly, with haplotype 1 assembled to chromosome level and haplotype 2 to scaffold level. The methods and results are clearly presented, and assembly quality is evaluated using multiple standard metrics. No annotation results were provided. Both the raw sequencing data and the assembled haplotypes have been made publicly available.  Overall, this study presents a high-quality genomic resource that will be useful for future comparative and evolutionary studies.

 In the abstract, the authors state that “Gene annotation of this assembly by Ensembl identified 13,139 protein-coding genes.” However, the gene prediction is not described elsewhere in the manuscript. While the primary aim of this Genome Note is not to present genome annotation, I would suggest that the authors briefly describe the gene prediction approach in the Methods section and report the number of predicted protein-coding genes in the Results section.

 To my understanding, the genome size of *Lasiommata paramegaera* appears to be somewhat larger than the average for *Nymphalidae*. Although detailed repeat annotation is beyond the scope of this study, a brief estimate of total repeat or transposable element content would be interesting to include, as it could indicate whether an elevated proportion of transposable elements contributes to the observed genome size.

 I have no major comments on the manuscript. My only minor comment concerns Figure 5: the red colour is not visible in the figure, and as a result the hap-1-only curve cannot be distinguished.

Are the datasets clearly presented in a usable and accessible format, and the assembly and annotation available in an appropriate subject-specific repository?

Partly

Are sufficient details of the sequencing and extraction, software used, and materials provided to allow replication by others?

Yes

Are the rationale for sequencing the genome and the species significance clearly described?

Yes

Are the protocols appropriate and is the work technically sound?

Yes

Reviewer Expertise:

Bioinformatics

I confirm that I have read this submission and believe that I have an appropriate level of expertise to confirm that it is of an acceptable scientific standard.

# Reviewer report: Reviewer response for version 1

**Published:** 2026-09-14

**Authors:** Automated Benchmarking

**Affiliations:** 1 Wellcome Sanger Institute, Hinxton, UK

As this article is a Genome Note, Automated Benchmarking has been used to determine whether the reported assembly passes three defined checks established by the Earth BioGenome Project: the raw and assembled data are publicly available, the Earth BioGenome Project three-part metric is met, and the BUSCO score passes the stated threshold. For Lasiommata paramegaera (ilLasPara1), all three checks passed. Data available: pass. ENA study PRJEB82399, raw-read accessions ERR13957086 and ERR13947560, and assembly accessions GCA_964656145.1 and GCA_964656155.1 are public. Earth BioGenome Project three-part metric: pass. Haplotype 1 is reported as 6.C.Q66, meeting the 6.C.Q40 reference standard. BUSCO: pass. Haplotype 1, which is proposed as the reference assembly for this species, has 98.5% complete BUSCOs (98.2% single-copy and 0.3% duplicated). BUSCO software version: 5.5.0; lineage dataset: lepidoptera_odb10. It meets the target of greater than 90% complete and single-copy BUSCOs. This decision uses values reported in the Genome Note and public accession records.

Reviewer Expertise:

NA

I confirm that I have read this submission and believe that I have an appropriate level of expertise to confirm that it is of an acceptable scientific standard.
